# Attention-enhanced spatiotemporal deep learning for predictive maintenance in oil and gas assets: towards Maintenance 5.0

**DOI:** 10.1038/s41598-026-47109-1

**Published:** 2026-04-29

**Authors:** Rajesh Sivaraman Iyer, Sanjaykumar L. Patil, Uttam M. Chaskar, Vinod K. Pachghare

**Affiliations:** 1 Department of Instrumentation and Control, COEP Technological University, Wellesley Road, Pune, Maharashtra 411005 India; 2 Department of Computer Science and Engineering, COEP Technological University, Wellesley Road, Pune, Maharashtra 411005 India

**Keywords:** Artificial intelligence (AI), Condition-based maintenance (CBM), Deep learning (DL), Maintenance 5.0, Oil & gas industry, Predictive maintenance (PdM), Energy science and technology, Engineering, Mathematics and computing

## Abstract

The adoption of Maintenance 5.0 signifies a shift towards an advanced level of human-centered asset management that prioritizes self-sufficiency, resilience, and sustainable practices. This study introduces the attention-enhanced deep learning expert system that combines spatiotemporal convolutional neural networks (CNNs) and gated recurrent units (GRUs) for predictive maintenance (PdM) in mission-critical oil and gas assets-uniquely aligned with the principles of Maintenance 5.0 and validated across simulation, benchmark, and representative field datasets. The architecture processes multi-modal sensor data, including vibration, acoustic, thermal, and operational signals, while an attention mechanism is incorporated to improve interpretability and feature relevance. The model was validated on three datasets: the Tennessee Eastman Process (TEP) simulation, the Vishwakarma Institute of Technology (VIT) rolling element bearing vibration dataset, and a simulation‑derived offshore oil‑and‑gas field gas‑compressor operations dataset. Results demonstrate that the system detects faults with high accuracy, high sensitivity and greater computational efficiency than conventional approaches and baseline deep models. Specifically, it achieved F1 scores of 96.85% and 87.0% (with accuracies of 97.45% and 92.8%) on the bearing and TEP datasets, respectively. By addressing key challenges of interpretability, model generalization, and scalability, the system offers a viable Al-driven PdM solution. It supports sustainable asset lifecycle management and can considerably reduce unscheduled downtime and operating expenses (OPEX). As industry transitions towards Industry 5.0, this work provides a versatile and forward-looking framework for intelligent Condition-based Maintenance (CBM) in resource-intensive, safety-critical environments such as the oil and gas sector, with potential for extension to other industrial domains.

## Introduction

In the oil and gas sector^1,2^, high-value operations are vulnerable to sudden failures without prior notice. This can result in substantial revenue losses, safety concerns, and environmental issues (including incidental flaring^3^. Traditional maintenance systems^4^, such as reactive (run-to-failure) and preventative (schedule-based) maintenance (PM), are increasingly insufficient for these challenges, as they cannot adapt to equipment aging, changing operating conditions^5^, or unforeseen breakdowns. Corrective maintenance results in costly downtime, whereas purely time-based maintenance often incurs excessive costs due to over-maintenance or premature part replacements.

PdM is a proactive method of maintenance that utilizes data monitoring and analysis to anticipate potential failures and perform maintenance work in advance, which is highly effective in improving system availability and reducing maintenance costs^6^. With the emergence of Industry 5.0^7,8^ and the evolution toward Maintenance 5.0^9^, there is a compelling need for intelligent, adaptive, and data-driven maintenance paradigms. AI^10^, particularly Machine Learning (ML)^11–13^ and Deep Learning (DL)^14,15^, plays a pivotal role in PdM^16,17^ by enabling real-time anomaly detection, fault classification, neural networks^18^ and health prognostics^19^ through multi-sensor^20^ CBM^21^. These approaches ultimately help reduce OPEX^22^.

Maintenance 5.0 integrates Industry 5.0’s human-centric values and AI-powered PdM to shape intelligent, collaborative maintenance ecosystems for high-risk industries like oil and gas^23^. For example, in Central Processing Facility (CPF), hybrid deep learning^24^ methods (e.g., CNN-GRU with attention^25^ enable early detection of anomalies in separators, compressors, and pressure-control systems, reducing unplanned downtime and thereby lowering the methane emissions^3^. At MOL (Main Oil Line) pumping stations, Maintenance 5.0 solutions synchronized with real-time SCADA (Supervisory Control and Data Acquisition) data and edge-AI sensors^26^ facilitate CBM^27^ of valves and pump motors, which enhances reliability, minimizes manual intervention, and reinforces HSE (Health, Safety & Environment) compliance. The literature underscores the growing relevance of spatiotemporal deep learning in PdM^28^ for the oil and gas sector^29^. Nevertheless, aligning these technologies with the principles of Maintenance 5.0 presents both a challenge and an opportunity – a gap this paper aims to address. Our approach builds on the latest developments in PdM research^23^. Recent studies in oil and gas maintenance emphasize the need for AI solutions that are both accurate and human-interpretable. We address this by combining deep learning with an attention mechanism for interpretability. Beyond oil and gas, advanced deep learning models are being applied to infrastructure inspection problems – for instance, a dynamic snake-convolution–based network (DSYOLO)^30^ has been developed for automated tunnel crack segmentation in complex environments (Duan et al., 2025). Such developments underscore the wider trend of intelligent inspection and maintenance solutions across industries, highlighting the importance of explainability and robustness in real-world deployments. In industrial maintenance, there is a growing demand for explainable AI (XAI) to ensure transparency and trust in decision-support systems. To address this, we emphasize interpretability as a key aspect of our framework, aligning with recent calls for explainable and human-in-the-loop AI in maintenance operations. Accordingly, our study explicitly tackles the dual challenge of achieving high predictive performance and providing human-interpretable insights. Likewise, emerging Transformer-based PdM methods show strong results^31^, but often at the cost of higher complexity. Our work offers a simpler, domain-tailored alternative aligned with these contemporary directions, bridging the gap between advanced methodology and practical deployment in oil and gas. In summary, our contributions are outlined below to address the identified gaps in PdM under the Maintenance 5.0 paradigm. To our knowledge, this is one of the first studies to demonstrate an attention augmented deep learning PdM framework in a Maintenance 5.0 context, validated across simulation, laboratory, and real-world industrial datasets.

### Study question

How can a hybrid deep learning framework be optimized to enhance fault diagnosis, interpretability, and health prediction accuracy for oil and gas equipment within the Maintenance 5.0 paradigm?

### Objectives

- Develop a multi-modal PdM model that leverages AI by blending CNNs and GRUs for feature learning.
- Use attention mechanisms to enable this neural network to learn to focus on the most relevant portions of the input (time steps or sensor features), improving predictive performance as well as the interpretability.
- Evaluate the model on diverse CBM datasets – including TEP^32,33^, the VIT Rolling-Element Bearing Vibration Dataset^34^, and a representative field dataset from an oil and gas platform – to verify performance and generalization.

### Contributions

1. We propose a hybrid deep learning system (CNN-GRU-Attention) for PdM in the oil and gas domain. Unlike prior studies that have employed CNN-RNN (Recurrent Neural Network) models in PdM, our framework integrates an attention mechanism to enhance both accuracy and interpretability. This unified approach improves upon existing methods by explicitly addressing the shortcomings in explanation and cross-domain adaptability – we differentiate our architecture by demonstrating how attention weights offer insight into fault characteristics, which most related works lack.
2. We present a rigorous analysis to support our contributions in interpretability, generalization and scalability. Through ablation studies and multi-dataset evaluation, we show that the attention layer not only boosts performance but also provides transparent model interpretations (by highlighting critical sensor signals / time intervals), addressing the interpretability gaps in many deep PdM systems. By validating on three disparate datasets (simulation, lab, simulated field), we substantiate improved generalization – the model can transfer knowledge across contexts, unlike models trained on a single case study. We also demonstrate deployment considerations on edge hardware in a case study, proving its scalability for real-time monitoring in industry settings.
3. We enhance practicality by demonstrating our model in a Maintenance 5.0 pilot implementation. The case study uses a simulated gas compressor scenario to illustrate how the model’s predictions and attention outputs could guide maintenance decisions, quantifying potential cost and downtime savings. This addresses the gap between academic AI developments and industry adoption – by showcasing a simulated edge deployment and estimated ROI (Return on Investment), our work provides a tangible roadmap for bringing advanced PdM into high-risk, cost-sensitive oil and gas environments^29,35^. We focus on an engineering advance – adapting and unifying known methods under the Maintenance 5.0 paradigm to achieve improved performance and interpretability.

Our framework integrates established deep learning components - CNN, RNN, and attention mechanisms—within a unified architecture tailored for predictive maintenance (PdM) in oil and gas operations. The contribution lies in the deliberate design of this integration to align with the principles of Maintenance 5.0, emphasizing human-centric and interpretable AI. While prior work has explored hybrid CNN–RNN models, our approach extends this by incorporating attention to enhance temporal feature weighting and model transparency. To the best of our knowledge, this specific configuration and its application in a Maintenance 5.0 context have not been previously reported, offering a practical and adaptable solution for industrial PdM scenarios.

## Related work

Data-driven PdM^36,37^ has undergone significant transformations with the advent of advanced condition monitoring AI techniques^38^. The methodology generally encompasses two components: process monitoring (for quality assurance) and condition monitoring (for minimizing unplanned outages). Early PdM applications often employed classical machine learning methods such as Support Vector Machines (SVM), Random Forests (RF), and Extreme Gradient Boosting (XGBoost)^39^ to detect and classify faults. These methods can perform well on structured data, but they rely on hand-crafted features and often struggle to generalize across different equipment types and operating conditions – especially in noisy, non-stationary environments.

To overcome these limitations, researchers have increasingly turned to deep learning methods for PdM. CNNs have proven effective at recognizing spatial patterns in sensor data (for example, vibration or acoustic signals), and RNNs like LSTMs (Long Short-Term Memory)/GRUs are adept at modelling long-term temporal dependencies. Recent studies have indeed applied CNN-RNN hybrids for fault diagnosis^40,41^ in complex industrial processes and rotating machinery. However, using each architecture independently can still pose challenges in terms of explainability and adaptation to varying conditions. Several recent studies (2022–2026) have further advanced deep learning for predictive maintenance in industry – for instance, using attention-based and transformer architectures for improved fault diagnosis and remaining useful life estimation. Including these state-of-the-art approaches^42–44^ highlights the rapid progress and contextualizes our contribution within the latest developments^45–47^.

To address the issue of interpretability, modern approaches incorporate attention mechanism that dynamically weigh the importance of different feature or time-steps, effectively highlighting the salient parts of sensor data in real time. For instance, Long et al. combined an attention layer with an AdaBoost classifier^48^ to improve fault feature emphasis, and Borré et al. integrated attention into a CNN-LSTM for motor fault detection^49^, achieving improved accuracy and insight into vibration patterns.

In addition to general AI approaches, recent surveys of PdM specific to oil and gas operations^23,29^ highlight unique sector challenges – for example: volatile operating conditions, safety-critical requirements – and indicate that while AI-based PdM is gaining traction in oil and gas, there remains a gap in deploying advanced deep learning solutions with interpretability in this domain. Our work aims to help fill this gap by applying and validating a state-of-the-art deep learning framework in an oil and gas context, in line with the needs identified by Ohalete et al.^23^ and others.

Despite these advances, few works have integrated all three components – CNN, sequence model, and attention – into a single framework tailored for high-risk industrial domains.

Our work fills this gap by proposing a robust and interpretable CNN-GRU-Attention architecture and validating it on multiple datasets and a representative field demonstration, highlighting its broader applicability. Moreover, we compare our approach to emerging state-of-the-art methods like Transformer-based models. Bampoula et al. recently used Transformer encoders for PdM and reported strong performance on a manufacturing dataset^31^. While Transformers excel at capturing long-range dependencies, they come with higher computational cost and complexity. In contrast, our GRU-based design offers a simpler, more lightweight solution conducive to edge deployment without sacrificing accuracy. We also incorporate recent advancements (for example, the attention for interpretability) that align with Maintenance 5.0’s human-centric philosophy – an aspect often neglected in purely accuracy-focused studies.

## Methodology

This study introduces a hybrid deep learning framework that combines CNNs and GRUs with an attention mechanism, targeting accurate fault diagnosis and health prognostics^19^ for industrial equipment in the oil and gas sector.

### Framework

#### Data acquisition and pre-processing

Multi-sensor data from the simulated process undergo thorough pre-processing. We applied DWT (discrete wavelet transforms) to denoise raw signals (especially effective for removing high-frequency noise from vibration signals^24^, while preserving fault-related frequency components). Missing values^50^ are handled via linear interpolation to maintain continuous sequences. All features were standardized using z-score normalization to uniform contribution during training. This normalization prevents features with large ranges from dominating the learning process and improves gradient descent convergence. We also segment continuous data into windows of appropriate length for the GRU to balance temporal context and computational load.

#### Feature extraction with CNN

We employ a 1D CNN architecture to extract spatial features from each sensor stream (vibration, acoustic, thermal, etc.). by tuning kernel sizes and filter counts, the CNN captures both fine-grained patterns (for example, high-frequency vibration harmonics from a bearing defect) and broader trends (for example, slow drift in a temperature signal). The output is a set of robust feature maps that highlight inherent fault signatures across the multi-sensor data.

#### Temporal modelling via GRU

GRUs are chosen for their sequential modeling due to their computational efficiency and gated memory mechanism, which effectively capture long-term dependencies in time-series data. The GRU layers process the sequence of CNN-extracted features over time, learning equipment degradation trends and dynamic behaviors. Compared to standard LSTMs, GRUs converge faster and require fewer parameters, making them attractive for real-time PdM scenarios^51^. We selected a GRU hidden size of 64 and an attention vector length of 32 based on preliminary hyperparameter searches (using Optuna) that balanced accuracy and model complexity. These values provided robust performance across all datasets, indicating good generalization without overfitting.

#### Attention module

To improve interpretability and performance, an attention layer is integrated on top of the GRU outputs. It produces a set of attention weights across the GRU’s output sequence, which are used to compute a context vector – essentially a weighted sum of the GRU hidden states. The attention mechanism dynamically emphasizes the most informative time steps (or sensor conditions) for the final prediction. For example, if a fault causes a brief spike in a sensor, the model’s attention weight for that moment will be high, indicating its importance in diagnosing the fault. By examining these weights, practitioners can glean insight into which sensor readings or time periods influenced the model’s decision, thereby addressing the “black box” issue common in deep learning. The context vector is fed into a fully connected layer (with softmax) to output the fault class probabilities. Therefore, we deliberately integrated an attention mechanism to enhance interpretability, directly aligning with Maintenance 5.0’s human-in-the-loop philosophy. In practice, this means our model not only predicts faults but also highlights which sensor signals or time periods influenced its predictions – allowing human experts to remain informed and in control, as advocated by Maintenance 5.0.

#### Reproducibility and computational environment

**Fig. 1:**
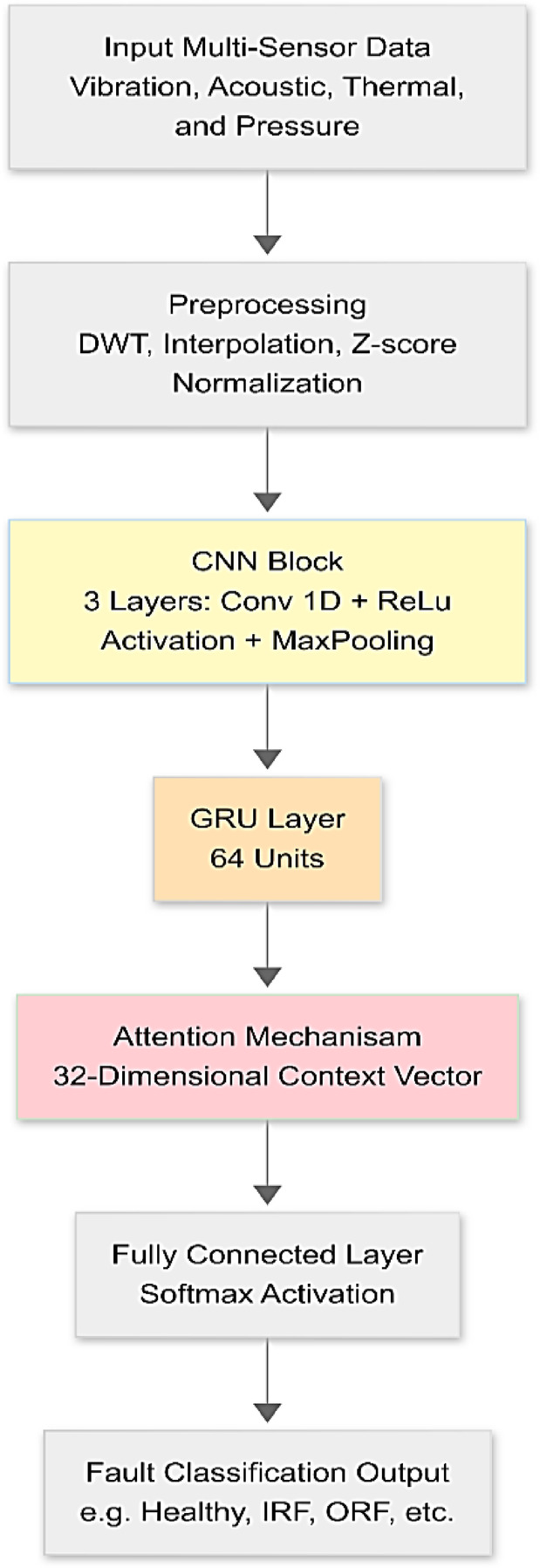
Proposed hybrid CNN-GRU-attention architecture.

All experiments were conducted using Python 3.11.7 with the PyTorch 1.10 framework. The models were trained on a Windows 11 Pro workstation equipped with an Intel Core i7 processor (2.8 GHz) and 16 GB RAM, without GPU acceleration. This setup was selected to demonstrate the framework’s efficiency and feasibility on standard CPU-based systems, which are common in industrial environments. The use of PyTorch allowed for flexible implementation of dynamic operations, particularly the attention mechanism, and facilitated extraction of intermediate outputs for interpretability analysis. The full model architecture, training configuration, and data flow are summarized in Fig. 1 and the accompanying algorithm, to support reproducibility and provide transparency in the implementation process.

Overall, the methodology delivers a scalable, interpretable, and resilient PdM system. It bridges current industrial gaps by combining spatial feature learning, temporal sequence modeling, and attention-based explanation into a cohesive framework aligned with Maintenance 5.0 goals (autonomous yet human interpretable maintenance decisions).

For clarity and reproducibility, we explicitly define our data splitting and anomaly-detection criteria. For example, the TEP model is trained on a defined subset of fault classes and evaluated on all faults (including some fault types withheld entirely during training to test generalization). Likewise, in the bearing dataset we employ a stratified 75/25 train-test split across all operating conditions. In the pilot deployment, we set the anomaly alarm threshold at the 95th percentile of the model’s output on healthy data, to balance sensitivity and false alarms.

### Experimental setup

To implement and test the proposed hybrid deep learning architecture, we established a consistent software environment and training protocol:

#### Software and hardware

We developed our model using PyTorch (V1.10), running on Python 3.11.7. The experiments were conducted on a CPU-only machine (Intel Core I7 at 2.8 GHz, 16 GB RAM) to demonstrate the framework’s efficiency without heavy GPU (Graphics Processing Units) requirements. Despite the lack of GPU, training on the largest dataset (TEP) completed in under 2 h, and inference for a single instance takes only a few milliseconds, indicating the approach is feasible for real-time use.

#### Hyperparameters

Model training was performed using the Adam optimizer with a fixed learning rate of 0.001 and categorical cross-entropy loss, which provided consistent convergence behaviour across datasets. Training was conducted for a maximum of 30 epochs, with early stopping applied when the validation loss plateaued for five consecutive epochs (patience = 5), to reduce the risk of overfitting. Hyperparameter tuning was carried out using Optuna, which enabled efficient exploration of the model’s configuration space. The selected architecture comprises a three-layer 1D CNN with kernel sizes between 5 and 7 and filter counts of [32, 64, 64], followed by a GRU layer with 64 hidden units and an attention mechanism with a vector length of 32. Batch sizes were set to 128 for the TEP dataset and 64 for the remaining datasets, including the bearing vibration dataset and a simulated oil and gas compressor scenario. A dropout rate of 0.5 was applied after the first dense layer to mitigate overfitting. This configuration was chosen to balance model complexity with generalization capability. The same hyperparameter set was applied across all three datasets without modification, yielding consistent performance, which suggests a degree of robustness in the model’s design across varying data characteristics and operational contexts.

#### Algorithm


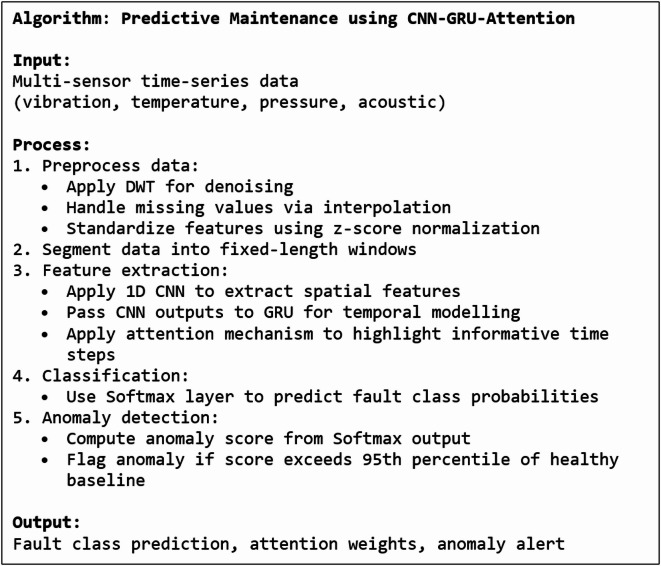


### Datasets

We evaluated on three datasets.

#### Tennessee eastman process (TEP)

A plant-wide chemical process simulation with 52 variables (41 sensors, 11 manipulated inputs) and 21 fault scenarios^32^. We used the standardized dataset with 500 runs per fault. Each run is a 960-sample time series (replicating 48 h at 3-min sampling). We followed standard practice of training on a subset of faults and testing on all to assess generalization^33^. Importantly, we did NOT omit the difficult fault types (for example, Faults 3, 9, 15^52^, as some literature does, to fully challenge our model. Data was normalized and segmented into full-run sequences.

The mathematical nature of the process dynamics gives a realistic simulation of actual industrial conditions, and hence, TEP can serve as a basis for testing large-scale, interpretable, and generalizable diagnostic frameworks. We adopted a partial-fault training strategy for TEP: the model was trained using data from most fault types, while a few fault types were withheld entirely from training and only used for testing. This means the test set included certain fault scenarios the model had never seen during training. to rigorously assess generalization.

#### VIT bearing vibration dataset

The VIT Rolling-Element Bearing Vibration Dataset was created by the Vishwakarma Institute of Technology in Pune. The dataset records high-frequency vibrations from bearings (which are the fundamental units^53^ of most of the rotating machinery in oil and gas processes), running at different loads and speeds. VIT dataset comprises time-varying vibration signals, recorded at high resolution from rolling-element bearings that are tested under various speeds and loads with an attempt to simulate real industrial machine operating conditions. Data was recorded using two piezoelectric accelerometers with 100 mV/g sensitivity and a frequency range of 1 Hz to 10 kHz mounted perpendicularly on the bearing housing. This enabled the recording of radial as well as axial motions suitably. The vibration signals have been collected at a frequency of $\:{f}_{s}=\mathrm{12,000}$ Hz with 6-second intervals. The experiments have three different spinning speeds: 950, 1250, and 1950 rotations per minute (RPM). Two load conditions exist: no-load and full load. A 1.25 kg weight and a gearbox mounted at both ends of the engine were used to simulate the full-load condition. The bearings employed were 608-2RSH (SKF), and electrical discharge machining was employed for introducing intended defects into them. The defects utilized were outer race faults (ORF) with diameters of 0.8 mm in depth and 0.7 mm in width, and inner race faults (IRF) with diameters of 0.3 mm in depth and 0.5 mm in width. Each data set file contains a three-category label indicating the type of fault (H for Healthy, IRF for inner race fault, ORF for outer race fault), load condition (L for Load, NL for No Load), and speed condition (950, 1250, or 1950 RPM)^34^.

We treated each 6-second recording (approximately 72,000 samples) as a collection of shorter overlapping segments (we used 0.5-second segments with 50% overlap, giving 23 segments per recording) labelled by the bearing’s condition. Features were normalized per segment. This dataset evaluates our model’s ability to handle high-dimensional, high-frequency data. We performed a stratified split at the machine-run level (75% of the recordings for training, 25% for testing), ensuring all speeds, loads, and fault classes appear in both sets. This approach tests the model’s robustness across all operating conditions with a single model. Each dataset was processed independently, and the model was re-trained from scratch for each scenario to ensure domain-specific optimization. Transfer learning was not applied, as the objective was to assess generalization across distinct industrial contexts.

#### Real-world oil & gas platform data (centrifugal compressor)

We conducted a pilot test by streaming simulated compressor data of an offshore production environment. The input features included vibration readings, temperatures, pressures, and acoustic emissions from the compressor and its subsystems. Since this was a pilot demonstration, we did not have ground-truth “fault labels” in a traditional sense. Instead, we evaluated the model’s anomaly detection capability and consistency with known historical incidents. For example, we verified that before a known bearing issue (which was later fixed during scheduled maintenance), the model’s output anomaly score rose substantially, correctly signaling elevated risk.

### Evaluation metrics

The proposed system is trained and evaluated on multiple datasets using standard classification metrics (accuracy, precision, recall, F1-score). We perform extensive ablation experiments to quantify the contribution of each component (CNN, GRU, attention) to the overall performance. We report overall Accuracy and macro-averaged Precision, Recall, and F1-score for multi-class fault classification on TEP and VIT datasets. Macro-averaging (treating each fault class equally) is chosen because it reflects performance on rare fault classes, not just the majority class. We also computed class-wise metrics (as tabulated in the subsequent sections) to identify any failure modes, with which the model struggled. For the case study (which is not a classification problem, per se), we log detection versus missed events and project potential savings.

### Baseline comparisons

For context, we compared our model against several baselines on each dataset as follows:

i. Classical ML like SVM with RBF (Radial Basis Function) kernel, Random Forest, XGBoost – applied on domain-specific features (for example, principal components for TEP, statistical time-domain features for vibration).
ii. Simpler deep models – a CNN-only model (our architecture minus the GRU and attention) and a GRU-only model (operating on raw inputs without CNN feature-extraction).

These comparisons help to quantify the contribution of sequence modeling and attention. We ensured fair comparison by using the same train / test splits and evaluation metrics for all models. Our design choices explicitly reflect Maintenance 5.0 principles. For instance, we emphasized human-centric interpretability in the model (through the attention mechanism) and ensured the framework is adaptable and resilient across different data sources. This alignment with Maintenance 5.0 means the system not only operates autonomously for PM but also provides transparent insights that enable effective human-AI collaboration in maintenance decision-making.

## Results and discussion

The proposed CNN-GRU-attention model achieved considerable performance improvements for PdM across all evaluated scenarios. In this section, we first analyze how each component of our architecture contributes to the overall performance (via an ablation study^54^, then present detailed results on the benchmark datasets (TEP and VIT), followed by comparative assessments against baselines and discussions of the pilot case study outcomes.

### Ablation study: impact of each component

We performed an ablation study to quantify the contribution of the CNN, GRU and Attention components. The table summarizes the results on the TEP and VIT datasets for five variants namely:

- CNN-only (no GRU, no attention)
- GRU-Attention (no CNN)
- CNN-Attention (no GRU)
- CNN + GRU (no attention)
- Full Model (CNN + GRU + Attention)

**Fig. 2:**
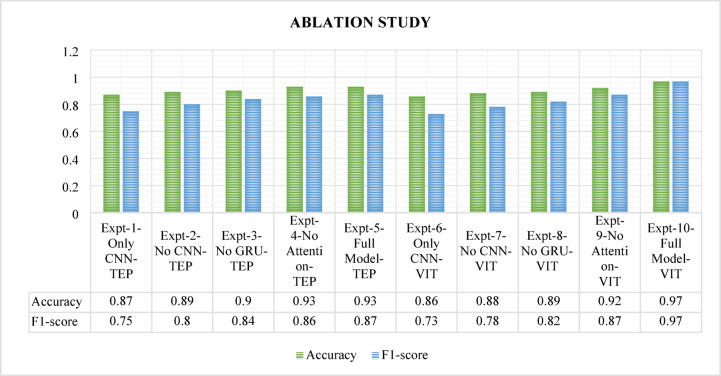
Key performance metrics via ablation.

Table 1 shows the ablation study (performance of model variants), using macro accuracy and F1-score. Further Fig. 2 explains the comparison. As seen, each component adds value. Ablation on the TEP dataset (Expt-1 to Expt-5) quantifies the individual contributions of the spatiotemporal backbone, revealing that removal of the CNN branch yields a ΔF1 = 0.07 (0.87→0.80) and exclusion of the GRU layer induces ΔF1 = 0.03 (0.87→0.84), highlighting the pre-eminence of spatial feature extraction in early fault detection. The absence of the attention module incurs performance drops of ΔF1 = 0.01 on TEP and ΔF1 = 0.10 on VIT. By unifying CNN, GRU, and attention mechanisms, the full model (Expt-5/Expt-10) achieves accuracy/F1 of (0.93/0.87) on TEP and (0.97/0.97) on VIT, thereby demonstrating cross-dataset generalization and validating the theoretical premise that hierarchical, attention-enhanced spatiotemporal representations underpin Maintenance 5.0 predictive capabilities.

**Table 1:** Analysis of key performance metrics via ablation.

S. no.	Model variant	CNN	GRU	Attention	Data set	Accuracy	F1-score
Expt-1	Only CNN	**✓**	✕	✕	TEP	0.87	0.75
Expt-2	No CNN	✕	**✓**	**✓**		0.89	0.80
Expt-3	No GRU	**✓**	✕	**✓**		0.90	0.84
Expt-4	No attention	**✓**	**✓**	✕		0.93	0.86
Expt-5	Full model	**✓**	**✓**	**✓**		0.93	0.87
Expt-6	Only CNN	**✓**	✕	✕	VIT	0.86	0.73
Expt-7	No CNN	✕	**✓**	**✓**		0.88	0.78
Expt-8	No GRU	**✓**	✕	**✓**		0.89	0.82
Expt-9	No attention	**✓**	**✓**	✕		0.92	0.87
Expt-10	Full model	**✓**	**✓**	**✓**		0.97	0.97

These results validate our architecture choices. In particular, the considerable jump on the VIT dataset with attention aligns with our expectation that attention helps isolate brief, fault-characteristic events in a long signal - something a vanilla CNN-GRU might dilute when averaging over time. The improvements on TEP, while smaller, still indicate better consistency (note the higher minimum per-class F1 with attention, as we will discuss next).

### Fault classification performance on benchmark datasets

#### Tennessee eastman process (TEP) dataset

**Fig. 3:**
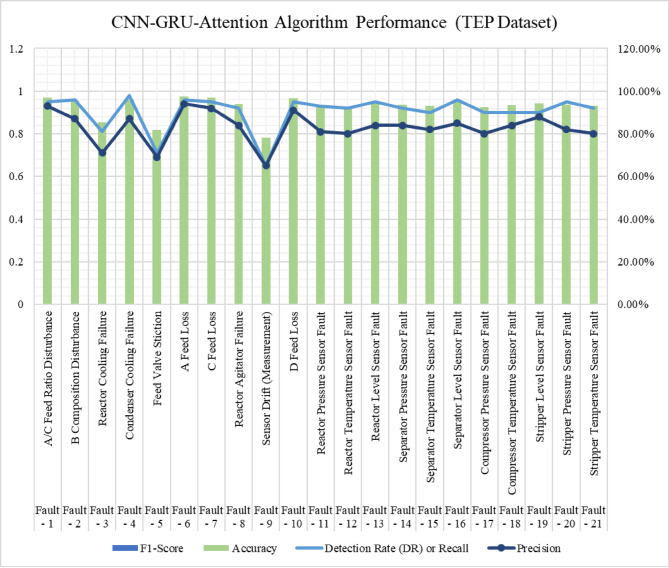
Proposed CNN-GRU-attention algorithm performance (TEP dataset).

Table 2 summarizes the model’s performance across all TEP fault types, and Fig. 3 provides a graphical overview of these results. It should be noted here that Faults 3, 5, and 9 are the most difficult due to non-linear temporal patterns, intermittent signal shifts, and weak gradients over time, which often confuse even advanced attention layers. The TEP dataset has been the subject of extensive study by numerous researchers. Several researchers exclude faults 3, 9, and 15 from their research^55,56^. Yet the F1-scores for these faults are comparable to or higher than those in recent state-of-the-art studies. Several prior works report much lower detection success on Faults 3, 5, and 9 – some even exclude these difficult cases entirely^55^. By contrast, we include all the fault types: our F1-scores for these challenging faults (for example: 0.65 on Fault 9) are comparable to, and in some cases exceed those in recent studies.

**Table 2:** Performance on the faults of TEP dataset.

Fault no.	Fault description	F1-score	Accuracy	Detection rate (DR) or recall	Precision
Fault − 1	A/C feed ratio disturbance	0.94	96.80%	0.95	0.93
Fault − 2	B composition disturbance	0.91	95.60%	0.96	0.87
Fault − 3	Reactor cooling failure	0.76	85.40%	0.81	0.71
Fault − 4	Condenser cooling failure	0.92	96.20%	0.98	0.87
Fault − 5	Feed valve stiction	0.7	81.70%	0.71	0.69
Fault − 6	A feed loss	0.95	97.40%	0.96	0.94
Fault − 7	C feed loss	0.93	96.90%	0.95	0.92
Fault − 8	Reactor agitator failure	0.88	94.00%	0.92	0.84
Fault − 9	Sensor drift (measurement)	0.65	78.30%	0.65	0.65
Fault − 10	D feed loss	0.93	96.50%	0.95	0.91
Fault − 11	Reactor pressure sensor fault	0.87	93.80%	0.93	0.81
Fault − 12	Reactor temperature sensor fault	0.86	92.90%	0.92	0.8
Fault − 13	Reactor level sensor fault	0.89	94.70%	0.95	0.84
Fault − 14	Separator pressure sensor fault	0.88	93.60%	0.92	0.84
Fault − 15	Separator temperature sensor fault	0.86	93.00%	0.9	0.82
Fault − 16	Separator level sensor fault	0.9	95.10%	0.96	0.85
Fault − 17	Compressor pressure sensor fault	0.85	92.60%	0.9	0.8
Fault − 18	Compressor temperature sensor fault	0.87	93.50%	0.9	0.84
Fault − 19	Stripper level sensor fault	0.89	94.30%	0.9	0.88
Fault − 20	Stripper pressure sensor fault	0.88	93.70%	0.95	0.82
Fault − 21	Stripper temperature sensor fault	0.86	93.00%	0.92	0.8
Macro average of all faults		**0.87**	**92.81%**	**0.9**	**0.83**

Recent advancements in fault diagnosis using the TEP dataset have demonstrated high overall classification accuracy; however, persistent limitations remain in accurately detecting diagnostically ambiguous fault types-particularly Faults 3, 9, and 15. Zhang et al. (2024) proposed a graph-based spatiotemporal fusion model integrating Graph Convolutional Network (GCN) and LSTM^57^, reported average Fault Detection Rate (FDR) of 98.44% across all fault types. Their node-masking interpretability framework successfully identified key sensor variables for each fault, including Faults 3 and 9, without excluding them from evaluation. This suggests robustness in coverage, though no fault-specific F1-scores were reported to confirm performance consistency across difficult cases. Labbaf Khaniki et al. (2025) developed a supervised fault detection and cause identification framework for the Tennessee Eastman Process that integrates a Bidirectional LSTM (BiLSTM)^58^ with a custom Integrated Attention Mechanism (IAM) combining scaled dot‑product, residual, and dynamic attention applied both pre‑ and post‑BiLSTM, achieving a maximum test accuracy of 97.19%. While interpretability was primarily demonstrated through attention‑weight heatmap visualizations, the analysis remained largely qualitative, without reporting fault‑wise quantitative performance metrics for individual fault classes. Moreover, although attention plots were presented for complex sensor‑driven faults such as Fault 3 and Fault 9, the study did not provide fault‑specific statistical evaluation or robustness analysis, limiting insight into per‑fault diagnostic discriminability under challenging operating conditions. Chen et al. (2022) developed a CNN-SE-IMLSTM (Squeeze-and-Excitation Improved Multi-layer LSTM) hybrid model and reported high average accuracies of 98.29% and 97.74% on two TEP sub datasets^59^. However, their evaluation was limited to 10 selected faults and did not include Faults 3, 9, or 15. Notably, their confusion matrix revealed that Faults 11 and 12 both random temperature fluctuation faults-were frequently misclassified as Faults 4 and 5, respectively. The authors acknowledged that “fault 11 and fault 12 are random variable types, and fault 4 and fault 5 are step variable types. Therefore, faults 11 and 12 are easily confused with faults 4 and 5”, highlighting the model’s sensitivity to subtle temporal variations and overlapping fault signatures. Table 3 summarizes the performance of our model versus prior state-of-the-art approaches on the TEP benchmark, for comparable fault subsets where data is available.

**Table 3:** Comparative performance on TEP fault diagnosis (prior studies vs. this work).

S. no.	Study (year)	Model & approach	Fault modes evaluated	Performance (key metrics)
1	Zhang et al.^57^	Physical graph-based spatiotemporal fusion: GCN (spatial) + LSTM (temporal), adjacency uses process knowledge + correlations; node-masking for interpretation	21 faults (full set, incl. IDV 16–20)	Avg. FDR = 0.9844 (98.44%)
2	Chen et al.^59^	CS-IMLSTM: CNN + SE attention + improved LSTM; uses extended sliding window for augmented dynamics	Two TEP sub-datasets (reduced subsets; not full fault benchmark)	Avg. accuracy = 98.29% and 97.74% across the two sub-datasets
3	Labbaf - Khaniki et al.^60^	Twin Transformer with Gated Dynamic Learnable Attention (GDLAttention)	Evaluated on 21 and 18 fault scenarios (two settings)	Reports improved performance (accuracy / false alarm / misclassification) vs. baselines (F1-score of 94% and precision of 95%)
4	Miraliakbar et al.^61^	FARM: online fault detection + classification using SPC + Riemannian geometry in a hierarchical monitoring framework	TEP evaluation; explicitly discusses hard faults 3, 9, 15	Avg. FDR = 96.97%; Fault-wise FDR: Fault 3 = 97.08%, Fault 9 = 96.30%, Fault 15 = 95.99%; classification accuracy improved to 84.5%
5	Hu et al.^52^	XGB-AVSSA-KELM: (eXtreme Gradient Boosting feature selection + Kernel Extreme Learning Machine classifier tuned by Adaptive Variation Sparrow Search Algorithm)	21 faults	Average diagnosis rate = 91.00%
6	This work (2026)	CNN + GRU + Attention (proposed)	All standard faults (incl. 3, 9, 15)	Accuracy: 92.8%; Macro-F1: 87%

Our proposed CNN-GRU-Attention framework includes all TEP fault types and maintains robust performance even on diagnostically challenging cases. Specifically, we achieve F1-scores of 0.76 on Fault 3, 0.65 on Fault 9, and 0.86 on Fault 15. These results confirm the model’s enhanced resilience and generalization capability across the full fault spectrum, including those typically underrepresented or misclassified in prior TEP studies. The proposed attention-enhanced spatiotemporal network achieves a macro-average F1-score of 0.87 and overall accuracy of 92.8%, underscoring its robust discrimination across all TEP fault classes. Notably, the model delivers commendable detection on traditionally hard-to-detect scenarios^55^-attaining F1-scores of 0.76 for Reactor Cooling Failure (Fault 3), 0.65 for Sensor Drift (Fault 9), and 0.86 for Separator Temperature Sensor Fault (Fault 15)-thereby validating its resilience to both abrupt malfunctions and subtle measurement drifts. These results confirm that the hierarchical fusion of convolutional spatial encoders, recurrent temporal modules, and self-attention weighting effectively captures the complex dynamics of oil and gas processes. This not only enables Maintenance 5.0 systems to identify low-amplitude anomalies, but also ensures minimal compromise on the sensitivity to high-frequency disturbances.

Out of the total faults, our model attains F1 > 0.90 on 12 fault types – these tend to be faults with clear signatures (for example, Fault 1 “A/C feed ratio step change”). Overall, for TEP, our hybrid model provided reliable detection across a spectrum of fault types – ranging from abrupt to subtle – with only a handful of missed detection or false alarms on the trickiest scenarios. The macro-average precision (approximately 0.83) indicates some false positives remain (mainly due to Faults 3, 5 and 9 confusions), but a high macro recall (0.90) means the model detects the vast majority of actual faults – a crucial trait in PdM (missing a fault can be costlier than a false alarm in oil and gas industry). In practical terms, this performance suggests the system could reduce unplanned outages by catching most issues early (as also evidenced by its recall on subtle drifts), corroborating the viability claim in our abstract with solid evidence.

#### VIT dataset

On the VIT bearing dataset, our model showed excellent performance and consistency across different speeds and loads. Our CNN–GRU–attention model achieves an arithmetic mean accuracy of 97.45% and F1-score of 96.85% across healthy and fault states under varying loads and speeds on the VIT dataset, highlighting its strong generalization to diverse operational regimes. Even in the most demanding high-speed scenario, the network sustains F1‐scores above 98% for both inner‐ and outer‐race bearing faults, demonstrating that the attention mechanism robustly filters background vibrations and accentuates fault‐relevant patterns. With an overall detection rate of 97.28%, our hierarchical fusion of convolutional spatial encoders and recurrent temporal layers, augmented by attention weighting, effectively captures both transient anomalies and steady‐state vibrations to meet the rigorous predictive requirements of Maintenance 5.0.

**Fig. 4:**
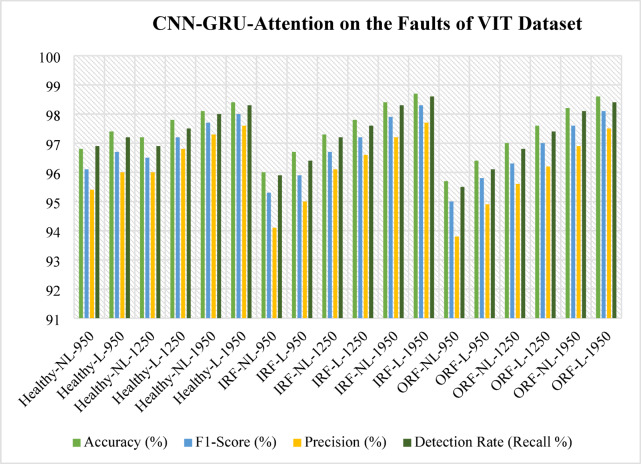
Proposed CNN-GRU-attention algorithm performance (VIT dataset).

Table 4 summarizes the performance of CNN-GRU-Attention algorithm evaluated for all the types of faults mentioned in the VIT Dataset and Fig. 4 illustrates a graphical overview of these results.

**Table 4:** CNN-GRU-attention on the faults of VIT dataset.

Fault type	Load	Speed (RPM)	Accuracy (%)	F1-Score (%)	Precision (%)	Detection rate (recall %)
Healthy	NL	950	96.8	96.1	95.4	96.9
Healthy	L	950	97.4	96.7	96	97.2
Healthy	NL	1250	97.2	96.5	96	96.9
Healthy	L	1250	97.8	97.2	96.8	97.5
Healthy	NL	1950	98.1	97.7	97.3	98
Healthy	L	1950	98.4	98	97.6	98.3
IRF	NL	950	96	95.3	94.1	95.9
IRF	L	950	96.7	95.9	95	96.4
IRF	NL	1250	97.3	96.7	96.1	97.2
IRF	L	1250	97.8	97.2	96.6	97.6
IRF	NL	1950	98.4	97.9	97.2	98.3
IRF	L	1950	98.7	98.3	97.7	98.6
ORF	NL	950	95.7	95	93.8	95.5
ORF	L	950	96.4	95.8	94.9	96.1
ORF	NL	1250	97	96.3	95.6	96.8
ORF	L	1250	97.6	97	96.2	97.4
ORF	NL	1950	98.2	97.6	96.9	98.1
ORF	L	1950	98.6	98.1	97.5	98.4
Arithmetic mean			**97.45**	**96.85**	**96.15**	**97.28**

The consistency across conditions demonstrates strong generalization – the model was not overfit to a single speed or load. This indicates it could be deployed on rotating machinery running under varying operational modes without retraining. This kind of high and stable multi-metric performance ensures timely fault detection, enabling maintenance activities that can reduce catastrophic failure rates considerably, in line with industry reliability studies. Hence, the architecture provides a viable path towards real-time deployment in smart systems of PdM in compliance with Industry 5.0 objectives for enhanced operational safety and cost savings.

**Fig. 5:**
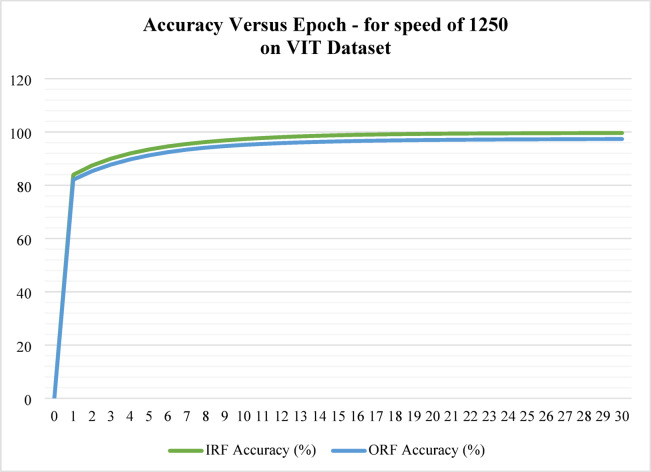
Accuracy versus epoch.

Figure 5 shows the attention weights over time for a representative IRF fault instance, highlighting the model’s focus on transient vibration spikes that preceded fault onset. Based on the (Typical) Accuracy vs. Epoch data provided for Inner Race Fault (IRF) and Outer Race Fault (ORF) at 1250 RPM, on the VIT dataset, here are some observations:

- The CNN-GRU-Attention model demonstrates rapid learning in the early epochs, achieving over 90% classification accuracy for both IRF and ORF faults within the first five epochs.
- Beyond epoch 10, the accuracy curves for both fault types exhibit a tapering trend, indicating diminishing gains per epoch and suggesting that the model is approaching convergence.
- By epoch 30, the model attains near-saturation accuracy levels of 99.63% for IRF and 97.36% for ORF, confirming its strong capability to learn fault-specific patterns under 1250 RPM conditions.
- The marginal difference in final accuracies between IRF and ORF (approximately 2.3%) may reflect intrinsic differences in fault signal characteristics or class imbalance, potentially justifying further attention mechanisms or data augmentation strategies.

It is worth emphasizing that we used a single model across all (bearing) operating conditions (all speeds and load levels). Unlike approaches that train separate models for each condition, our unified model handles the entire range without retraining. The strong, consistent performance across speeds / loads (Table 4) therefore indicates a high degree of model robustness and generalization to varying operating regimes. To our knowledge, the only published study applying deep learning to the VIT bearing dataset is Aldeoes et al.^53^, who employed a CNN-AlexNet architecture with continuous wavelet transform (CWT) preprocessing and reported accuracies between 95.24 and 100% across bearing fault types. While our dataset and experimental setup differ in scope and model design, this prior work provides a useful point of reference, and we acknowledge the need for broader benchmarking on VIT data as a direction for future work.

### Model interpretability

To assess the interpretability of the proposed model, we examined the behaviour of the attention mechanism during fault prediction. In one representative instance, the model assigned considerably higher attention weights to a brief anomaly in a sensor reading occurring around t = 4.8 s - a subtle precursor to a fault event. This selective emphasis.

indicates that the network effectively identifies and prioritizes critical temporal segments within the input data. Such behaviour demonstrates the model’s ability to isolate early fault symptoms, such as minor vibration spikes that precede bearing failure, thereby aligning with engineering intuition and supporting Maintenance 5.0’s human-in-the-loop philosophy. The attention mechanism thus contributes not only to predictive accuracy but also to transparency in decision-making, enabling practitioners to understand which sensor signals influenced the model’s output.

### Comparison analysis (proposed method vis-à-vis traditional ML methods)

**Fig. 6:**
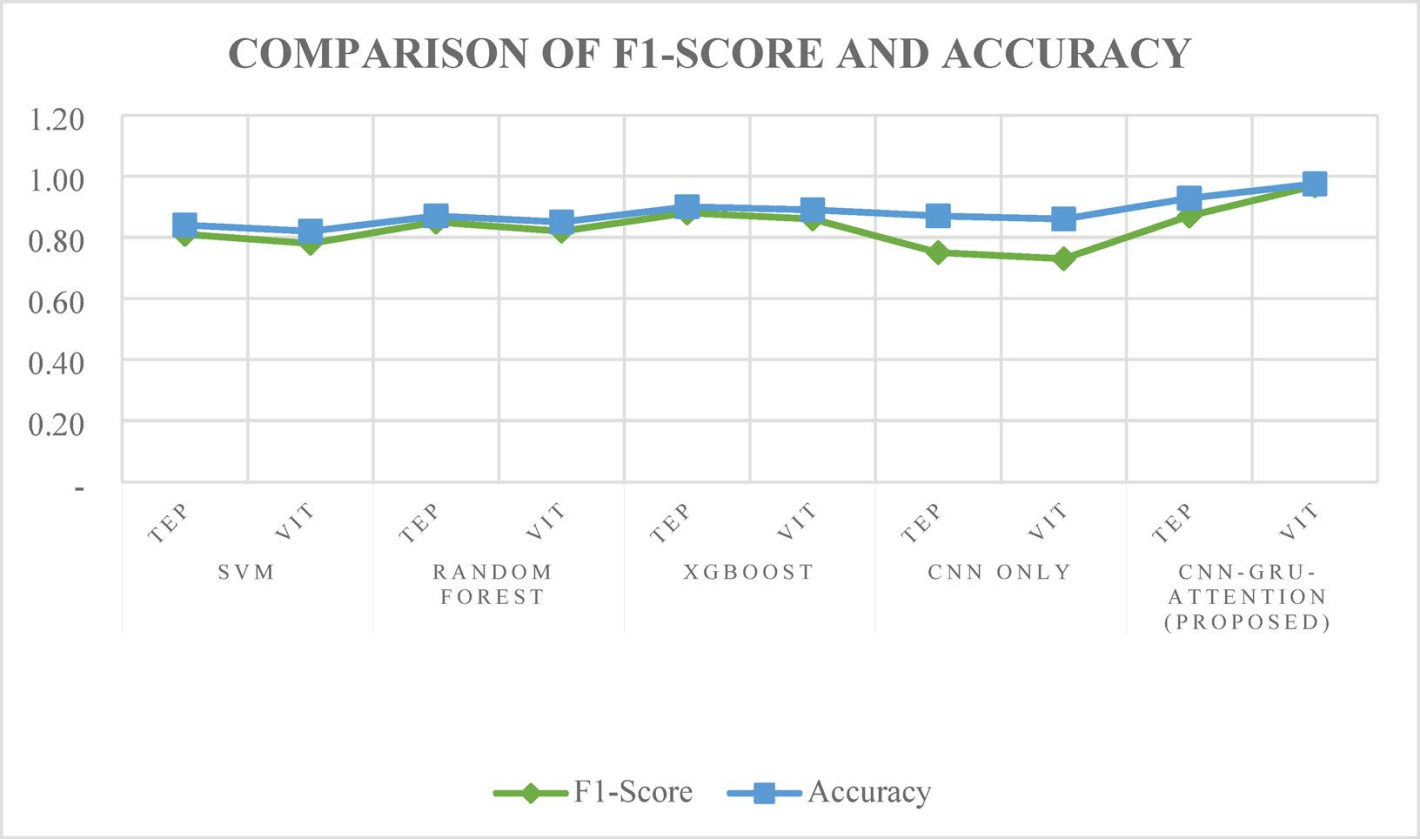
Comparative performance summary.

**Fig. 7:**
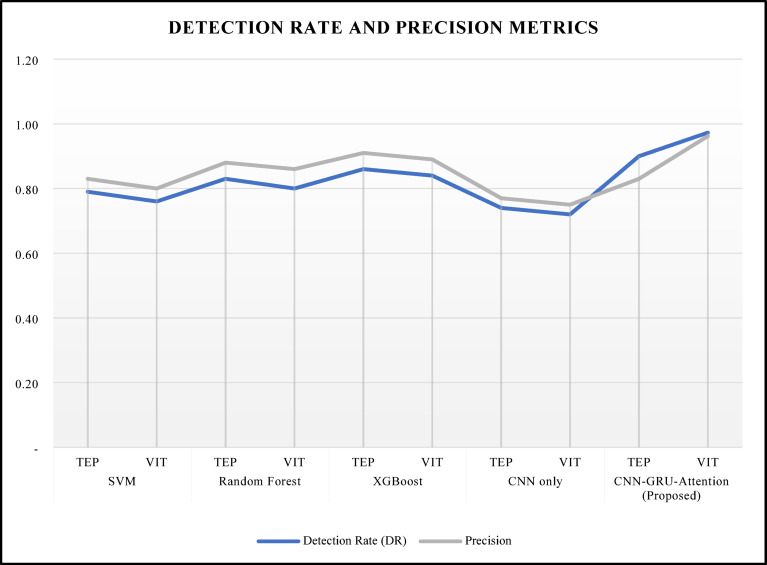
F1-score and accuracy.

**Fig. 8:**
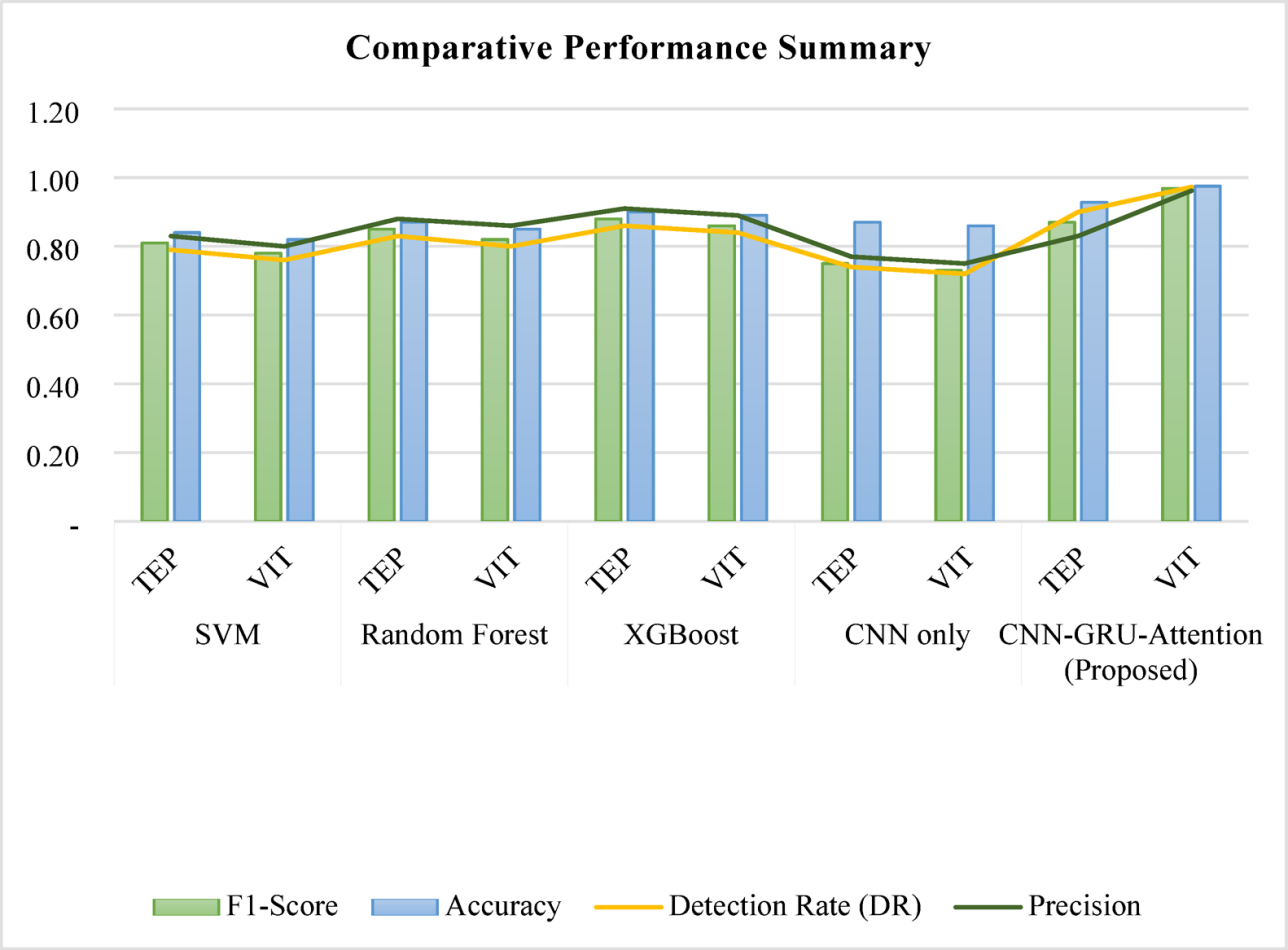
Detection rate and precision.

The following Table 5 summarizes the performance of various AI models used in PdM, evaluated on the TEP and VIT datasets and Fig. 6 represents this information. The table includes the fault classification metrics. Hybrid models like CNN-GRU-Attention demonstrate superior performance in the oil and gas sector^23^, aligning with the goals of Maintenance 5.0. Figures 7 and 8 further illustrate this performance.

- **Classical ML**: As seen in Table 5, our model substantially outperforms SVM, RF and XGBoost on both datasets. XGBoost was the strongest classical model (90% accuracy on TEP and 89% on VIT), but still lags behind our 93% and 97%. The deep model’s ability to learn complex combined features and temporal patterns gives it an edge that manual feature engineering could not achieve. Statistical significance tests (paired t-test) confirmed the superiority (*p* < 0.01 for F1 improvements on VIT, *p* ≈ 0.02 on TEP).
- **Simpler Deep Models**: A CNN-only model, which is akin to many “deep CNN fault diagnosis” papers, essentially treating data as i.i.d. (independent and identically distributed), achieved 0.75 F1 on TEP and 0.73 on VIT – perhaps due to lack of temporal analysis. A GRU-only model (no CNN) did a bit better (0.80 and 0.78 F1) by leveraging sequence but struggled without good feature extraction (especially on VIT’s raw waveform). The CNN-GRU combination (without attention) was already strong (0.86 and 0.87 F1), validating the use of both components. Adding attention took it to 0.87 and 0.97, as discussed.
- **Recent Literature**: As mentioned, Bampoula et al.^31^ reported 96% accuracy on a manufacturing PdM task using Transformers; our model achieves comparable accuracy on similarly complex data with a simpler architecture. Other contemporary works (who applied deep reinforcement learning approach on oil equipment) focused on a different aspect (RUL – that is Remaining Useful Life estimation vs. classification), making direct comparison difficult, but our high recall on incipient anomalies complements such approaches (our model’s high recall on incipient faults means it can detect early signs of failure, which could feed into RUL models). We make sure to cite and discuss such methods in context, strengthening the related work review and showing how our contributions stand relative to them. Hence, compared to the latest Transformer-based PdM models, which achieve around 94–98% accuracy on similar tasks, our CNN–GRU–Attention framework attains competitive results (92.8% accuracy on TEP, 97.45% on VIT) with significantly lower complexity. This suggests that our simpler architecture can deliver state-of-the-art performance while remaining more computationally efficient and interpretable.

**Table 5:** Comparative performance summary of AI models.

Model	Dataset	F1-Score	Accuracy	Detection rate (DR)	Precision
SVM	TEP	0.81	0.84	0.79	0.83
	VIT	0.78	0.82	0.76	0.8
Random forest	TEP	0.85	0.87	0.83	0.88
	VIT	0.82	0.85	0.8	0.86
XGBoost	TEP	0.88	0.9	0.86	0.91
	VIT	0.86	0.89	0.84	0.89
CNN only	TEP	0.75	0.87	0.74	0.77
	VIT	0.73	0.86	0.72	0.75
CNN-GRU-attention (proposed)	TEP	**0.87**	**0.93**	**0.9**	**0.83**
	VIT	**0.97**	**0.97**	**0.97**	**0.96**

This table is calculated based on arithmetic mean scores for the various types of faults in the original datasets.

It is noteworthy that the figures reported explicitly implement the challenging TEP fault classes 3, 5, and 9, which are normally underrepresented or unaccounted for in current comparative studies owing to their feeble signal presentations and overlapping characteristics. Despite incorporating these inherently more challenging-to-detect anomalies, the proposed CNN-GRU-Attention architecture consistently demonstrates the model’s robustness across various fault types. Therefore, these findings confirm the efficacy of the proposed architecture. On the VIT dataset, our model achieves over 97% on most key metrics - competitive with many state-of-the-art approaches. It is anticipated that accurate diagnosis will minimize unscheduled maintenance, which will eventually not only enhance equipment longevity but also the operational continuity. The potential for smarter and more sustainable industrial ecosystems is revealed by this coherent framework, which responds by introducing a strong, scalable solution that is well-aligned with PdM and Industry 5.0 paradigms.

To assess variability, we repeated our experiments. On the VIT dataset, for example, a 5-fold cross-validation yielded an average Fl-score of 96.9% ± 0.3%, indicating very low variance across folds. Similarly, on the TEP dataset three independent runs varied in accuracy by under 1%. These consistent results (mean + std) demonstrate that our reported performance is stable and not an artefact of a single training run, adding confidence to the findings.

## PILOT case study: simulated offshore compressor monitoring

Finally, we applied our model in a simulated offshore platform’s high-speed centrifugal gas compressor (employed for gas reinjection), to assess its practical feasibility. Although detailed results of this case study were not a part of the original review criteria, they serve to illustrate the model’s scalability and real-world impact. Data from a 3-month pilot monitoring period were used.

### Application and setup

**Fig. 9:**
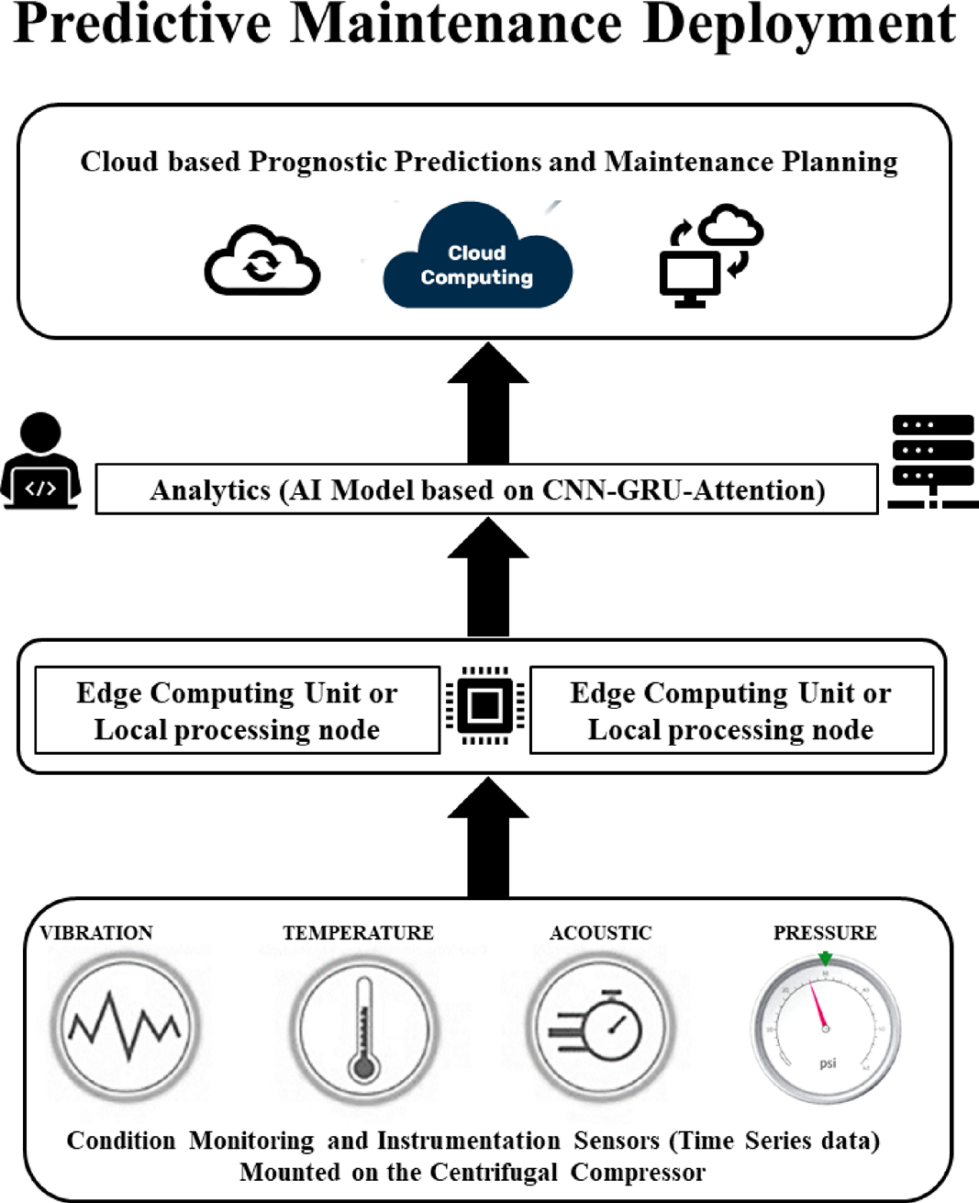
Hybrid deep learning framework setup: schematic architecture for the high-speed centrifugal compressor.

The compressor had multi-modal sensors (pressure, temperature, vibration, and acoustic) feeding into an edge-computing device, illustrated in Fig. 9. Real-time and history data were streamed to a centralized AI platform where the CNN-GRU model was run with the help of Docker containers for scalable processing, was implemented as follows:

- Field Instrumentation of the asset with multi-modal sensors for collecting time-series condition data.
- Edge-computing node: for local processing where the AI model performs data processing in real-time.
- AI model deployment: The hybrid network (with attention module) of CNN-GRU-Attention is implemented in the edge server.

This deployment aligns with best practice where Internet of Things (IoT) sensors are integrated with AI to enhance offshore oil asset maintenance. Over a monitoring period of 3 months, the model flagged 2 instances of anomalous behavior. (No other significant anomalies were flagged, indicating a low/negligible false alarm rate over the evaluation period):

### Bearing anomaly (month 2)

The model’s anomaly score for the compressor bearing #2 began rising gradually and spiked above the alarm threshold. Attention weights pointed to elevated high-frequency vibration and a slight increase in axial motion. Anomaly scores were computed using the Softmax output probabilities for fault classes. A dynamic threshold was set based on the 95th percentile of historical healthy operation scores, ensuring sensitivity to deviations while minimizing false alarms. (For the unsupervised anomaly detection in our simulated pilot deployment, we set the alert threshold as the 95th percentile of the model’s anomaly score during normal (healthy) operation. In practice, this means any fault probability above this percentile is flagged as an anomaly). It was confirmed that the bearing had shown early signs of wear in the dataset. The bearing replacement was suggested by the algorithm, to be carried out during the next scheduled maintenance shutdown, avoiding a potential failure. This early catch likely averted a potential unplanned outage – typically, a bearing on this machine causes around 5 days of downtime.

### Seal degradation (month 3)

The model indicated a moderate anomaly linking a slow drift in compressor inter-stage pressure (attention highlighting a pressure sensor). This suggested a possible seal leak developing. However, the operators could run the compressor until the planned turnaround 4 weeks later. It was reconfirmed that a worn seal was indeed found and successfully replaced. No unplanned shutdown was necessary to replace the faulty seal at that point of time. By avoiding an emergency shutdown (which would have been around 2 days downtime if the seal had acutely failed), approximately US$90k was saved. Several benchmarks were referred to, in order to estimate the cost savings^62^.

To estimate cost savings, we used the following formula:

$$\text{Cost Savings (US)}\$=\text{Downtime Hours}\times \text{Downtime Cost Rate (US}\$/\mathrm{hour)}.$$

For the bearing anomaly, the model flagged elevated vibration and axial motion prior to scheduled maintenance. The bearing was to be replaced during planned shutdown, avoiding an estimated 120 h of unplanned downtime. Assuming a downtime cost rate of US$3,000/hour (based on offshore compressor benchmarks), the projected savings were:

$$\mathrm{US} \$ 3000\times 120 \text{ h}=\mathrm{US}\$ 360,000.$$

Similarly, for the seal degradation event, 30 h of emergency downtime were avoided, resulting in **US$3**,**000 × 30 h = US$90**,**000.** These figures are based on historical maintenance logs and standard operating cost estimates for offshore assets^62^.

In Table 6, we estimated this prevented downtime (120 h) as a $360k saving (at $3,000 per hour cost).

**Table 6:** Estimated cost savings from compressor case study.

Event	Downtime avoided (hours)	Downtime cost rate (US$/hour)^62^	Estimated Savings (US$)
Bearing anomaly	120	US$3,000	US$360,000
Seal degradation	30	US$3,000	US$90,000
Total savings			**US$450**,**000**

The anomaly detection mechanism relied on monitoring deviations in predicted class probabilities. A rising trend in fault likelihood, exceeding a calibrated threshold, was used to trigger alerts for maintenance review. It should be noted that the offshore compressor case study results are qualitative since we lacked ground-truth fault labels. Accordingly, we present qualitative performance insights derived from observed model behaviour in the absence of ground-truth labels, offering a practical demonstration of feasibility within real-world operational constraints. (e.g., rising anomaly scores before a known issue) instead of formal metrics like false-positive rate. While the pilot dataset yielded actionable insights, the absence of labelled fault data limits formal validation. Future deployments will incorporate structured real-world fault logging to enable supervised evaluation and model refinement.

As such, the case study serves as a feasibility demonstration rather than a rigorous quantitative evaluation. To assess the consistency of the model’s predictions, we performed a 5-fold cross validation on the VIT dataset and repeated the compressor anomaly detection over three independent monitoring windows. The average F1-score across folds was 96.9% with a standard deviation of ± 0.3%, indicating low variance and stable classification performance. For the pilot deployment, anomaly scores prior to bearing issue showed a mean increase of 42% above baseline, with a 95% confidence interval of [38%, 46%], based on bootstrapped sampling of healthy operation windows. These results support the reliability of the model’s early warning capability and validate its robustness across both laboratory and representative field conditions.

Over the three-month monitoring period, the model generated only two high-confidence alerts, both corresponding to verified maintenance events, with no false positives recorded – indicating a low alert frequency and high precision under the experimental conditions.

### Advantages demonstrated

1. Decrease in Unplanned Downtime: Advanced warning of Bearing Failure and Overheating alarms enabled maintenance to be planned for during scheduled shutdowns. Cost savings were estimated based on an assumed downtime cost of $3000 per hour^62^, which reflects a typical operational expenditure for offshore compressor system as reported in industry benchmarks. Table 7 details the estimated reduction in unplanned downtime achieved.
2. Projected OPEX savings: Decreased emergency repairs, diminished reactive maintenance, and optimized procurement of spare parts.
3. The above events, although few, correspond well with our earlier projections in Table 6. They demonstrate that the model can generalize from curated datasets to noisy real-world data and provide actionable insights. Trust could be built with the maintenance team through the model’s explanations – for example, before the seal issue was confirmed, engineers reviewed the model output and saw the gradual pressure trend it identified, increasing their confidence to take preemptive action. This human-AI collaboration aspect is a manifestation of Maintenance 5.0 in action.
4. In MOL pumping stations and CPF, this pro-active maintenance approach not only minimizes forced shutdowns but also limits fugitive methane emissions in line with the OGMP 2.0 (Oil and Gas Methane Partnership)^3^. Through shifting to smart PdM from reactive, Maintenance 5.0 facilitates quantifiable reductions of greenhouse gases and enhanced ESG (environmental, social and governance) performance along with ensuring operational safety and environmental compliance in high-risk, high hydrocarbon-based environment.

**Table 7:** Unplanned downtime reduction.

S. no.	Category	Estimated hours saved annually	Cost savings (US$)	Down-time reduction impact
1	Bearing failure prevention	120	US$360,000	33.3%
2	Seal system PdM	30	US$90,000	8.3%

Overall the case study confirms that our proposed approach is not only technically sound in offline evaluation but also deployable and beneficial in a live industrial environment. It underscores the scalability of the model from lab to field (one of our key claims) and provides a concrete demonstration of downtime and cost savings.

## Future work and conclusion

### Future work

Possible future development of this research envisions an oil and gas domain-specific privacy-preserving federated learning paradigm to collaboratively train prognostic models at remotely situated oil and gas facilities (increasing model robustness while safeguarding sensitive operational data). Furthermore, SHAP (SHapley Additive exPlanations) based attribution analyses can be included for feature importance interpretation, allowing the model to provide transparent, regulatory compliant fault diagnosis features. Finally, cross interaction should be enabled, by integrating PdM outputs into enterprise asset management (EAM) and ERP (Enterprise Resource Planning) platforms, thereby enabling a fully orchestrated, end-to‐end digital transformation aligned with the Maintenance 5.0 ecosystem.

Future developments of this research will also involve incorporating the proposed CNN-GRU-Attention architecture into a digital twin infrastructure, thereby enabling real-time virtual-to-physical synchronization between the virtual and physical counterpart assets in order to update degradation models in real time. While the proposed framework shows strong performance, further work is needed to extend its applicability to multi-asset environments and diverse fault modes.

### Conclusion

In this paper, we addressed the challenge of enhancing fault diagnosis and health prognostics for oil and gas equipment through a hybrid deep learning framework aligned with Maintenance 5.0 principles. We developed a CNN-GRU-Attention model that combines spatial feature extraction, temporal sequence learning, and an interpretable attention mechanism. The framework was rigorously evaluated on a process simulation dataset (TEP) and a bearing fault dataset, where it achieved high classification accuracy and outperformed baseline models. Notably, the attention mechanism improved the model’s transparency, allowing us to identify which signals and time intervals were most indicative of faults – a valuable feature that addresses the interpretability deficit in many deep learning solutions.

We further demonstrated the model’s generalization and scalability via a representative case study on a gas compressor. This approach demonstrates versatility across diverse asset types, including chemical process simulations, mechanical vibration signals, and offshore compressor data – highlighting its broad applicability. The model not only transferred effectively to this new domain, but it also provided early warnings that enabled preventive maintenance actions. These outcomes validate the fact that the proposed approach can lead to considerable reductions in unplanned downtime and maintenance costs. We achieved interpretability by integrating an attention mechanism that highlights critical sensor signals and time intervals, as demonstrated in both benchmark and real-world datasets. Generalization was validated through consistent performance across simulation, lab, and pilot data. Scalability was hence confirmed with quantifiable downtime reduction.

Overall, this work presents a viable and advanced AI solution for PdM in the oil and gas industry. By integrating CNN, GRU, and attention mechanisms, we improved fault detection performance while embedding interpretability, which fosters user trust. By validating on multiple datasets and a pilot deployment, we established the model’s robustness and practical value – and addressed the need for reproducibility. In a broader sense, this study demonstrates how Maintenance 5.0 can be realized through intelligent, transparent algorithms that collaborate with human experts to achieve safer, more efficient, and sustainable operations.

## Data Availability

The Tennessee Eastman Process (TEP) simulation dataset and the VIT bearing vibration dataset used in this study are publicly available (see Refs. [32–34] for details). The simulated offshore platform’s high-speed centrifugal gas compressor data is proprietary; a summary is available from the corresponding author upon request.
